# Comparison of Selected Machine Learning Algorithms for Industrial Electrical Tomography

**DOI:** 10.3390/s19071521

**Published:** 2019-03-28

**Authors:** Tomasz Rymarczyk, Grzegorz Kłosowski, Edward Kozłowski, Paweł Tchórzewski

**Affiliations:** 1 University of Economics and Innovation in Lublin, 20-209 Lublin, Poland; tomasz@rymarczyk.com; 2 Research & Development Centre Netrix S.A., 20-704 Lublin, Poland; pawel.tchorzewski@netrix.com.pl; 3 Faculty of Management, Lublin University of Technology, 20-618 Lublin, Poland; e.kozlovski@pollub.pl

**Keywords:** machine learning, inverse problem, electrical impedance tomography, image reconstruction, industrial tomography

## Abstract

The main goal of this work was to compare the selected machine learning methods with the classic deterministic method in the industrial field of electrical impedance tomography. The research focused on the development and comparison of algorithms and models for the analysis and reconstruction of data using electrical tomography. The novelty was the use of original machine learning algorithms. Their characteristic feature is the use of many separately trained subsystems, each of which generates a single pixel of the output image. Artificial Neural Network (ANN), LARS and Elastic net methods were used to solve the inverse problem. These algorithms have been modified by a corresponding increase in equations (multiply) for electrical impedance tomography using the finite element method grid. The Gauss-Newton method was used as a reference to machine learning methods. The algorithms were trained using learning data obtained through computer simulation based on real models. The results of the experiments showed that in the considered cases the best quality of reconstructions was achieved by ANN. At the same time, ANN was the slowest in terms of both the training process and the speed of image generation. Other machine learning methods were comparable with the deterministic Gauss-Newton method and with each other.

## 1. Introduction

This article presents the results of research on the use of tomographic sensors for the analysis of industrial processes with the use of dedicated measuring devices and image reconstruction algorithms. 

Electrical impedance tomography (EIT) is a non-invasive, high-potential application imaging method. It is suitable for continuous real-time visualization of the dynamic distribution of electrical conductivity inside the tested object [1]. To perform EIT reconstructions, we use weak alternating currents (1–5 mA) with low frequency (1–100 kHz) and measure the appropriate peripheral voltages by means of a set of electrodes attached to the object’s surface [2]. A cross-sectional image of internal spatial conductivity is obtained from voltage measurements gained from different electrode pairs. Despite its relatively low spatial resolution, the EIT is now a widely accepted tomographic imaging technique that is widely used in many areas, such as monitoring industrial processes [3,4,5], geophysical research [6,7,8] and biomedical diagnosis [2,9,10]. Mathematical reconstruction of conductor maps in the EIT is about solving a non-linear and ill-posed inverse problem from noisy data [11]. Regulatory techniques can be used to mitigate the instability of solutions. One of the most commonly used methods is a one-step approach to Gauss-Newton reconstruction (GN) [12], which allows the use of sophisticated, regulated models to describe the problem of the inverse EIT through a heuristic determined predecessor [13]. Landweber iteration is a modification of the steepest gradient descent approach and is also widely used in EIT [14]. The algebraic reconstruction technique (ART) is a valid method of reconstructing the computed tomography images that can be used in the EIT [15]. Other important methods include: regularization using total variation (TV) [16], which allows image reconstruction while preserving the edge, split augmented Lagrangian shrinkage algorithm [17] and the generalized vector sampled pattern matching method (GVSPM) [18].

Because deep learning is good for mapping complicated nonlinear functions, attempts are increasingly being made to apply deep learning methods based on convolutional neural networks (CNNs) for EIT/ERT (electrical resistivity tomography) image reconstruction [11]. Among the CNN, the deep D-bar methods are also used [19]. D-bar methods are based on a rigorous mathematical analysis. They provide robust direct reconstructions by using a low-pass filtering of the associated nonlinear Fourier data [9].

In the EIT tomography, algorithms belonging to machine learning methods can be successfully used. Typical examples of this kind of method are: Lasso (least absolute shrinkage and selection operator), Elastic net, least-angle regression (LARS) [6], artificial neural networks [20] and convolutional neural networks [11], multivariate adaptive regression splines (MARS), k-nearest neighbors (KNN), random forest (RF), gradient boosting machine (GBM) [21], Principal Component and Partial Least Square Regression [22].

The current development of EIT algorithms is largely focused on the use of machine learning methods [23]. Hence the need to verify whether such algorithms are in fact better than the classical, known deterministic methods to which the Gauss-Newton method belongs [12,24].

In comparison to other known imaging methods used in industry [25], electrical impedance tomography (EIT) has a number of advantages. These include, among others: higher time resolution, lower costs, opportunities for wider use, etc. However, reconstruction of the EIT may be unstable and has a fundamental disadvantage resulting from the need to solve the inverse problem [26]. The sensitivity of the EIT solutions to measurement, numerical and model errors entails the need to adjust the model parameters to specific cases. Many such methods have been developed over the years. These serious constraints on the EIT therefore favor the development of more sophisticated algorithms [27,28,29]. It is worth mentioning that most 2D reconstruction methods are also applicable in 3D situations with minor modifications [30].

The authors of the article developed three original variants of known algorithms based on machine learning techniques, and then compared them to the deterministic method as well as to each other. In order to make a precise assessment enabling a reliable comparison, universal evaluation metrics were used: Mean Squared Error (MSE), Relative Image Error (RIE) and Image Correlation Coefficient (ICC).

Advanced automation and control of production processes play a key role in enterprises [31,32]. Technological equipment and production lines can be considered the heart of industrial production, while information technologies and control systems are its brain. Tomographic imaging of objects creates a unique opportunity to discover the complexity of the structure without the need to invade the object. There is a growing need for information on how internal flows behave in the process equipment. This should be performed non-invasively by tomographic instrumentation [33]. 

Sensor technologies are mainly based on electrical tomography (ET) [34,35,36,37,38], which includes electrical capacitance tomography (ECT) [39,40,41,42,43,44,45] and electrical resistance tomography (ERT) [7,46,47]. It allows reconstruction of the image by the distribution of conductivity or permittivity of the object from electrical measurements at the edge of the object. 

The results of the reconstruction of individual algorithms with different measurement models were compared. The tests were carried out for real data obtained from real laboratory measurements. The electronic devices for measuring the material values and to collect data from the measurement sensors were designed and made by the authors.

**Figure 1:**
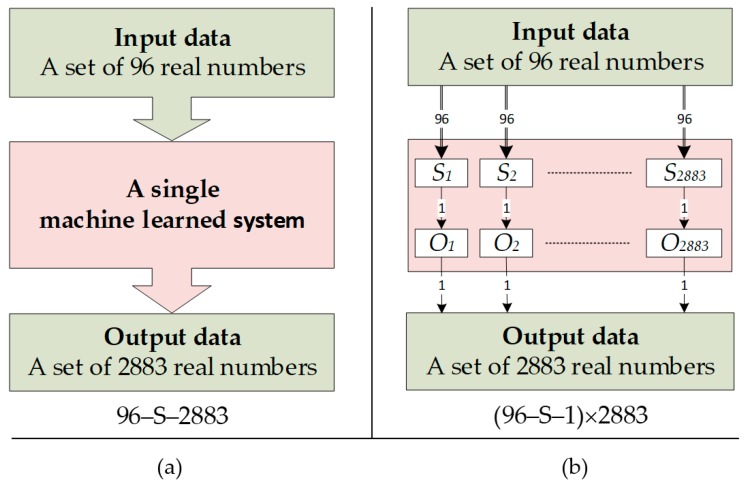
Comparison of the traditional concept with the improved concept: (**a**) a single prediction system with 96 predictors and 2883 responses; (**b**) multiple prediction system composed of 2883 separately trained subsystems, each of which has 96 predictors and 1 response.

The main novelty of the presented method is a machine learning approach based on learning many separate subsystems (ANN, LARS, Elastic net), while each subsystem is dedicated to a single pixel of the output image (Figure 1). The deterministic method, Gauss-Newton with Laplace regularization should be treated as a reference, enabling objective comparison of standard techniques with machine learning methods.

Figure 1a shows the traditional machine-trained algorithm. It consists of a singular predictive (regression) system with many outputs. The input vector includes 96 measurements of voltage drops measured on individual electrode pairs. The predictive system has 2883 outputs, which makes its training difficult. The large number of system outputs is the main reason for the unsatisfactory quality of reconstructed tomographic images.

Figure 1b shows the scheme of the novel multiple system. Its characteristic feature is that on the basis of the same 96-element vector of predictors, 2883 separate prediction subsystems (*S*_1_, *S*_2_,…, *S*_2883_) were trained. Each of the subsystems generates only one independent output (response), which is the value of a single pixel of the reconstructed image (*O*_1_, *O*_2_,…, *O*_2883_). Thanks to this approach, each pixel of the output image is the result of the operation of a single-output prediction subsystem. Subsystems with one output are easier to train than subsystems with multiple outputs. Thanks to this, the results obtained using the presented concept (96—S—1) × 2883 are better than those obtained using the traditional concept 96—S—2883. 

The article consists of four sections. The measurement models, machine learning methods and descriptions of algorithms were presented in Section 2. The results of the research work in the form of reconstruction of images for measurement data are shown in Section 3. In Section 4, the results obtained are discussed. It also summarizes the presented research.

## 2. Materials and Methods

This section presents the tomographic methods, process tomography, measuring devices, laboratory systems, mathematical algorithms and measurement models used in image reconstruction based on synthetic data and real measurements. Laboratory equipment, tomography devices designed at Research & Development Centre Netrix SA, the Eidors toolbox [48], Microsoft tools, Matlab, Python and R language were used during the research.

### 2.1. Electrical Tomography

Electrical tomography is an imaging technique that uses different electrical properties of different types of materials, including biological tissues. In this method, the power or voltage source is connected to the object, followed by the emergence of current flows or the distribution of voltage at the edge of the object. The collected information is processed by an algorithm that reconstructs the image. This tomography is characterized by a relatively low image resolution. Difficulties in obtaining high resolution result mainly from a limited number of measurements, nonlinear current flow through a given medium and too-low sensitivity of measured voltages depending on changes in conductivity inside the area. Electrical tomography has historically been divided into electrical capacitive tomography for systems dominated by dielectrics, and electrical resistance tomography. The basic theory can be obtained from Maxwell’s equations.

A complex “admittivity” can be defined as follows:

(1) γ=σ+iωε

where *ε* is the permittivity, *σ* is the electrical conductivity, and *ω* is the angular frequency. 

In the case of the electric field strength (**Ε**), the current density (**J**) in the test area will be related to Ohm’s law:

(2) J=γE

The gradient of the potential distribution (*u*) has the form:

(3) E=−∇u

Due to the fact that there are no sources from the Ampère law in the studied region, we have:

(4) ∇·J=0

Potential distribution in a heterogeneous, isotropic area:

(5) ∇·(γ∇u)=0

where u is the potential. 

Where the capacitance or resistance dominates, the equation factor should be simplified to the form:

(6) $$\nabla \cdot \left(\sigma \nabla u\right)=0\;for\;\frac{\omega \varepsilon }{\sigma }\ll 1\left(\mathrm{ERT}\right)$$

(7) $$\nabla \cdot \left(\varepsilon \nabla u\right)=0\;for\;\frac{\omega \varepsilon }{\sigma }\gg 1\left(\mathrm{ECT}\right)$$

By solving the inverse problem, we obtain the distribution of material coefficients in the studied area.

Electrical resistance tomography in a process tomography can be interchangeably called electrical impedance tomography (EIT). In the following part of this work, we will mainly use the name, EIT [49,50,51].

**Figure 2:**
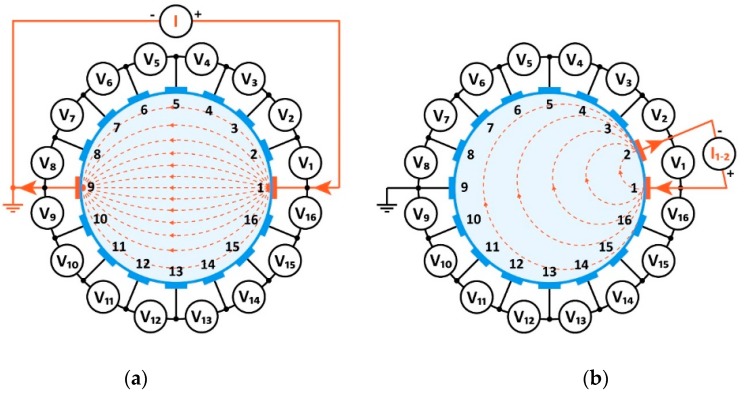
Measurement model in electrical impedance tomography: (**a**) opposite, (**b**) neighboring method.

The inverse method and neighboring method in EIT for collecting data from potential measurements at the edge of an object for 16 electrodes is shown in Figure 2.

### 2.2. Measurement Models

**Figure 3:**
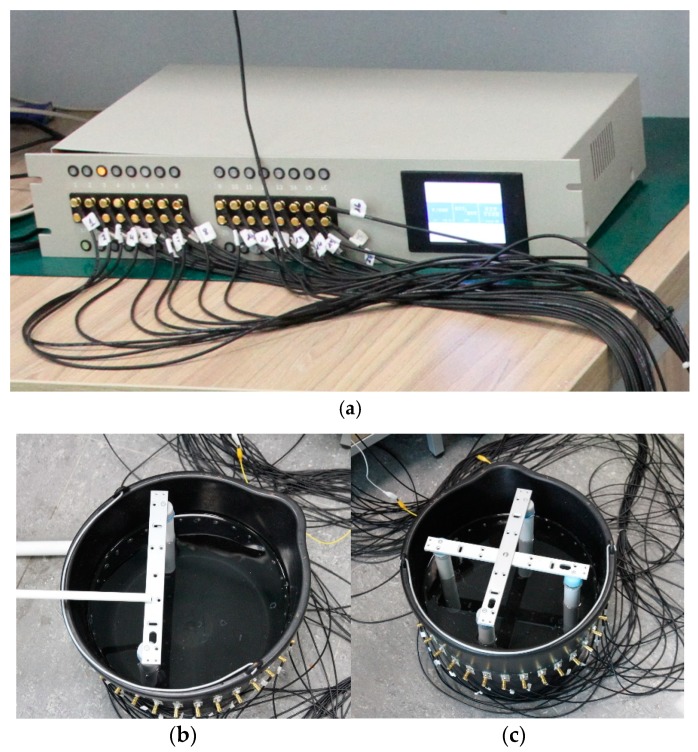
The test stand: (**a**) the measurement device—a hybrid tomograph made by the Netrix S.A. Research and Development Center, (**b**) tank with 2 phantoms, (**c**) tank with 4 phantoms.

In order to test the effectiveness of algorithms for the analysis of processes in industrial tomography, three real measuring models were implemented. Electrical tomography was implemented for the analysis. Figure 3a presents the EIT measuring device (hybrid tomograph), which was made by the Netrix S.A. Research and Development Center. A bucket with electrodes was used as the tank or industrial reactor model (Figure 3b,c).

**Figure 4:**
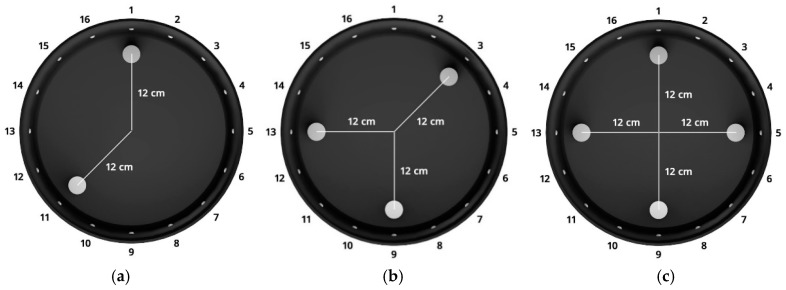
Three variants of the arrangement of phantoms in the tested tank with 16 electrodes: (**a**) 2 phantoms, (**b**) 3 phantoms, (**c**) 4 phantoms.

The arrangement of phantoms inside the investigated object is presented in Figure 4. This is a plan view that corresponds with the pictures of the tank shown in Figure 3.

**Figure 5:**
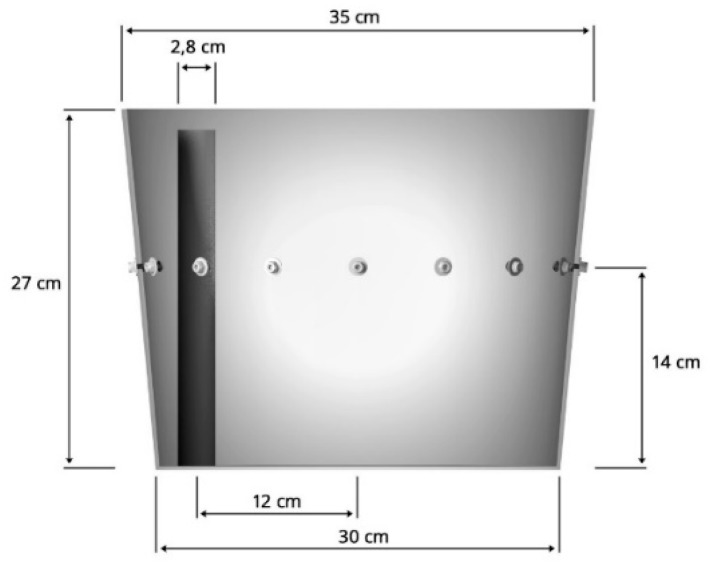
Dimensioned model of the EIT tested tank.

Figure 5 shows a side view of the dimensioned model of the EIT tested tank. On the left side, a tube immersed in the tank with its diameter can be seen.

Based on the above physical models, a special simulation algorithm was developed to generate learning cases used during the training process of the machine learning systems. Each training case was generated in accordance with the following procedure. First of all, we assume a homogeneous distribution of electrical conductivity. Then, we randomly select the number of internal inclusions. We assume that as a result of the draw we receive a maximum of five objects, each with a circular shape. The radius and conductivity are such that they correspond to the actual tests carried out by the EIT. During the next stage of calculations, the center of each internal object is drawn. For the obtained conductivity distribution, measurement voltages are determined using the finite element method.

**Figure 6:**
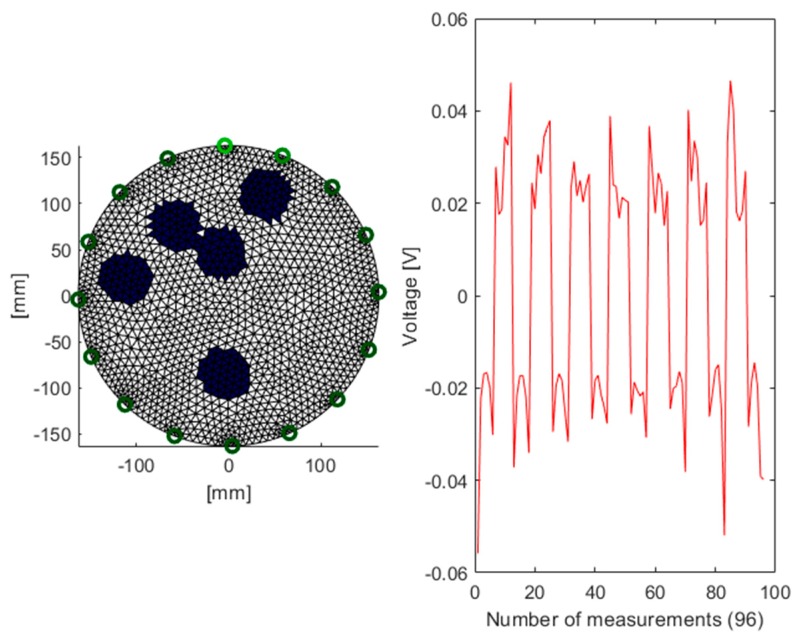
A training case generated with the simulation method with a graph showing the voltages.

Figure 6 shows one of the 50,000 generated cases used to train the predictive system. The cross section of the tank contains 5 randomly arranged artifacts, which corresponds to the 96 vector voltage measurements. Because the polarization of the electrodes changes during individual measurements, the voltage varies during the interval (−0.06; +0.06).

Based on the dimensions of the physical model, output images (reconstructions) for 3 cases of arrangement of the tubes were also generated (see Figure 4). The background pixel values are 1 and in the reconstructive images are marked in white. In turn, the spots (pixels) of the occurrence of the tubes have a value close to 0 and are colored dark blue.

Algorithm 1 shows the pseudo code used to generate training cases. The script generating the simulation data of the measurements on the electrodes included artificial noise (line *8* in Algorithm 1). For this purpose, a random number generator with a normal distribution was used, with an expected value of 0 and a corresponding standard deviation. In addition, the voltages determined on the basis of numerical simulation are always subject to a certain error, especially when, as in the described case, we make calculations on a grid consisting of a relatively small number of finite elements.

**Algorithm 1.** The pseudo code to generate learning cases*1*.N = 50000;% The number of cases*2*.**for** 1: N*3*. random selection of the number of objects;% set of NumberOfObjects variable*4*. **for** 1: NumberOfObjects*5*.  random selection of the object’s location;% center and radius*6*. **end***7*. adding an output image to the set of training cases;% saving response data*8*. determination of voltages and adding Gaussian noise;% Gaussian noise = randn(1, 96) × 5 × 10^−5^*9*. saving the values of voltages to the training set;% saving input data*10*.
**end**


### 2.3. Algorithms and Methods

There are many methods and algorithms used in optimization problems [52,53,54,55,56,57]. In this article, the authors chose deterministic algorithms based on the Gauss-Newton method as a reference to the machine learning methods. The Gauss-Newton method is often used in electrical tomography because it is quite effective. The next algorithms were based on machine learning methods [8,58], in which an innovative approach to tomographic problems was presented.

#### 2.3.1. Image Reconstruction

Process tomography also belongs to the problems of the inverse electromagnetic field. The inverse problem is the process of optimization, identification, or synthesis in which the parameters describing a given field are determined based on the possession of information specific to this field. Such issues are difficult to analyze. They do not have unambiguous solutions and are ill-conditioned due to too little or too much information. They are sometimes contradictory or linearly dependent. Knowledge of the process can make image reconstruction more resistant to incomplete or damaged data. The numerical analysis of the problem was carried out using the finite element method.

The colors of individual pixels on the image correspond to the conductance of the examined cross-section parts. An approach in which each of the separately trained subsystem generates only one output, that is, the value of a single pixel of the output image allows for better mapping of the values of electrical measurements.

To confirm the above thesis, a number of experiments were carried out using three neural networks differing in structure and number of outputs. Three types of ANN with the following structures were trained: 96—10—1 (96 predictors, 10 hidden neurons, 1 response), 96—10—10 (96 predictors, 10 hidden neurons, 10 responses) and 96—20—10 (96 predictors, 20 hidden neurons, 10 responses). The smaller the Mean Square Error (*MSE*) and the bigger the regression (*R*), the better is the quality of ANN. Responses (output pixels) were chosen randomly. The set of 50,000 cases was randomly divided into 3 subsets: training, validating and testing in the proportion of 70:15:15. The results of the experiments are presented in Table 1.

Only the testing set was used to assess the network quality. To increase the objectivity of experiments, the indicators in Table 1 ($\bar{MSE}$, $\bar{R}$) were the arithmetic mean of 10 experiments performed for each of the three types of ANN.

As you can see, the best results were obtained for the ANN with a single output (96—10—1). A more complex 10-output network (96—20—10) was better than the simpler 96—10—10 ANN having 10 neurons in the hidden layer. However, both neural networks with 10 responses turned out to be worse than ANN with a single response. The abovementioned tests proved that the variant of ANN with one output turned out to be the best. For this reason, in the research multiple LARS, Elastic net and ANN systems were used, in which each of the subsystems generated only one response value.

**Figure 7:**
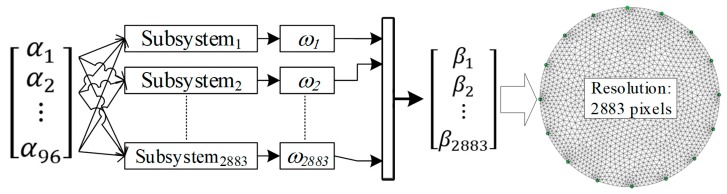
A mathematical neural model for converting electrical signals into images.

Figure 7 presents an outline of a machine learning system that was applied to all 3 methods: LARS, Elastic net and ANN. A distinguishing feature of the presented concept is the separate training of each of the 2883 machine learning subsystems. Their number is equal to the resolution of the image output grid (2883 pixels).

#### 2.3.2. Gauss-Newton Method

The Gauss-Newton method is an effective approach to solve inverse problem in the electrical impedance tomography. It is worth emphasizing that such a problem is nonlinear and ill-posed. In difference imaging, the Gauss-Newton method can be used to minimize differences between reference and inhomogeneous data [12,59].

In general cases, image reconstruction involves determining the global minimum of the objective function, which can be defined as follows:

(8) $$F\left(\sigma \right)=\frac{1}{2}\left\{\|U_{m}-U_{s}\left(\sigma \right){\|}^{2}+\lambda^{2}\|L\left(\sigma -\sigma^{*}\right){\|}^{2}\right\}$$

where:

**U***_m_*—voltages obtained as a result of the measurements
**U***_s_*(**σ**)—voltages received by numerical calculations (FEM) for given conductivity **σ**
σ*—conductivity represents known properties
*λ*—regularization parameter (positive real number)
**L**—regularization matrix.

Using appropriate approximations, it can be shown that the conductivity in the iteration denoted by *k* + 1 is given by the following formula:

(9) $$\sigma_{k+1}=\sigma_{k}+\alpha_{k}{\left(J_{k}^{T}WJ_{k}+\lambda^{2}L^{T}L\right)}^{-1}\left[J_{k}^{T}W\left(U_{m}-U_{s}\left(\sigma_{k}\right)\right)-\lambda^{2}L^{T}L\left(\sigma_{k}-\sigma^{*}\right)\right]$$

where: **W**—weighting matrix (it is usually a unit matrix), **J***_k_*—Jacobian matrix calculated in *k*-th step, *α_k_*—step length. The Gauss-Newton method with Laplace regularization was implemented in our research.

#### 2.3.3. LARS

Machine learning is related to the ability of the software to generalize based on previous experience. The important thing is that these generalizations are designed to answer questions about both previously collected data and new information. Using statistical methods with different regression models was presented in [60]. This approach enables quick diagnosis combining low cost and high efficiency. The selection of variables and the detection of data anomalies are not separate problems. To use the variables and outliers at the same time, the low angle regression (LARS) algorithm is used. While it is prudent to be cautious about the generalization of a small set of simulation results, it seems that LARS combined with dummy variables or row samples can provide computationally efficient, robust selection procedures. The proposed multiply LARS algorithm calculates all possible Lasso estimates for a given problem using an order of magnitude of less computing time. Another variation of LARS implements the linear regression of forward stagewise, this combination explains similar numerical results previously observed for Lasso and Stagewise and helps to understand the properties of both methods. A simple approximation of LARS degrees of freedom is available, from which the estimated prediction error value is taken.

If the regression data has only additional outliers, then we can start with a simple regression model:

(10) Y=Xβ+ε

where $Y\in R^{n},X\in R^{n\times \left(k+1\right)}$ denote the observation matrices of response and input variables, respectively, and $\beta \in R^{k+1}$ denotes the vector of unknown parameters. The object, $\varepsilon \in R^{n}$ presents a sequence of disturbances. The Least Angle Regression algorithm selects the subset of appropriate variables from entire set of available input variables. The linear model is built by employing the forward stepwise regression, where at each step the best variable is inserted to model. 

The algorithm of Least Angle Regression is applied as follows:

1. standardize input variables;
2. select the most correlated input variable with the output variable. Add input variable to the linear model;
3. determine the residual from the obtained model;
4. add a variable which is the most correlated with the residual to the model;
5. move coefficient *β* towards its least-squares coefficient;

Repeat steps 2–5 for the suitable number of iterations.

#### 2.3.4. Elastic Net

Elastic net is a regularized regression method that linearly combines the L1 and L2 penalties of the Lasso and ridge methods [61,62,63]. Lasso is a regularization technique. The implemented multiply method can be used to reduce the number of predictors in a regression model or it selects among redundant predictors. 

The equation is used to determine the linear regression:

(11) $$\underset{\left(\beta_{0},\beta^{\prime }\right)\in R^{k+1}}{\min}\frac{1}{2n}\overset{n}{\underset{i=1}{\sum }}{\left(y_{i}-\beta_{0}-x_{i}\beta^{\prime }\right)}^{2}+\lambda P_{\alpha }\left(\beta^{\prime }\right)$$

where $x_{i}=\left(x_{i1},\ldots ,x_{ik}\right)$, $\beta^{\prime }=\left(\beta_{1},\ldots ,\beta_{k}\right)$ for 1≤i≤n and $P_{\alpha }$ is an Elastic net penalty

$P_{\alpha }$ is defined as:

(12) $$P_{\alpha }\left(\beta^{\prime }\right)=\left(1-\alpha \right)\frac{1}{2}\|\beta^{\prime }{\|}_{L_{2}}+\alpha \|{\beta^{\prime }\|}_{L_{1}}=\overset{k}{\underset{j=1}{\sum }}\left(\frac{1-\alpha }{2}\beta_{j}^{2}+\alpha \left|\beta_{j}\right|\right)$$

We see that the punishment is a linear combination of norms $L_{1}$ and $L_{2}$ of unknown parameters $\beta^{\prime }$. The introduction of the parameter-dependent penalty function to the objective function reduces the estimators of unknown parameters.

#### 2.3.5. Multiply Neural Network

This chapter presents the neuronal model enabling efficient reconstruction of tomographic images. Effective use of multiply artificial neural networks in tomography is possible, but the effectiveness of this tool depends on many conditions. First of all, ANNs (artificial neural networks) are able to effectively visualize objects, many of which are already known. Each subsystem means one neural network. All neural networks were trained based on a set of 50,000 simulation-generated cases. 

A serious problem limiting the ability to generalize ANNs is overfitting. A good technique to reduce overfitting is to fundamentally limit the capacity of the model. These approaches are called regularization techniques. Among them, the following techniques can be distinguished: parameter norm penalties, early stopping, dropout, and transfer learning. In the case described, the technique of early stopping was used [64]. 

This technique tries to stop an estimator’s training phase prematurely, at the point where it has learned to extract all meaningful associations from the data, before beginning to model its noise. This is done by monitoring MSE (Mean Squared Error) of the validation set and terminating the training phase when this metric stops falling. This way, the estimator has enough time to learn the useful information but not enough to learn from the noise.

All cases were randomly divided into 3 sets: training, validating and testing in 70:15:15 proportions. The training set (35,000 cases) was used to properly train each of the subsystems. The validation set (7500 cases) was used to determine the moment of stopping the iterative training process. The condition for stopping the learning process was a non-decreasing MSE for the validation set for the next 6 iterations. The test set (7500 cases) can be used for independent assessment of network quality after the learning process (MSE, R). The structure of each of the neural networks can be described as follows: 96—10—1. This means that each ANN was a multi-layered perceptron with 96 predictors, one hidden layer with 10 neurons and the output layer with one neuron. Logistic functions were used as the activation functions. All ANNs were trained using the Levenberg-Marquardt algorithm.

Algorithm 2 in the form of Matlab code represents the iterative process of training the multiple neural network shown in Figure 5. In a single structural variable called *nets_for_pixels*, all 2883 neural networks were recorded.

**Algorithm 2.** The Matlab code for training multiple ANN system% $X^{\prime }$- input matrix 96 × 50000 of training cases% $Y^{\prime }$- output matrix 2883 × 50000 of training cases% Choose a Training FunctiontrainFcn = 'trainlm'; % In this case Levenberg-Marquardt backpropagation was chosenhiddenLayerSize = 10;  % Choose a number of hidden layersnet = fitnet(hiddenLayerSize,trainFcn);  % Create a fitting network under variable ‘net’% Choose input and output pre/post-processing functions% ‘removeconstantrows’ - remove matrix rows with constant values% ‘mapminmax’ - map matrix row minimum and maximum values to [−1 1]net.input.processFcns = {'removeconstantrows','mapminmax'};net.output.processFcns = {'removeconstantrows','mapminmax'};% Setup division of data for training, validation, testingnet.divideFcn = 'dividerand'; % Divide data randomlynet.divideMode = 'sample'; % Divide up every samplenet.divideParam.trainRatio = 70/100; % 70% of cases is allocated for trainingnet.divideParam.valRatio = 15/100; % 15% of cases is allocated for validationnet.divideParam.testRatio = 15/100; % 15% of cases is allocated for testingnet.performFcn = 'mse'; % Mean Squared Error will be used for performance evaluationx = *X^'^*;y = *Y^'^*;N=2883; % The resolution of output picture gridparfor i=1:N  % Start ‘for’ loop with parallel computing  % Assign an i-th row of reference cases to the variable t. Each of the 2883 lines corresponds   % to one pixel of the output image  t = y(i,:);  % Train the network. The variable ‘nets_for_pixels’ is a structure that consists of 2883   % separately trained neural networks.  [nets_for_pixels{i},~] = train(net,x,t);end % End ‘parfor’ loop

It should be emphasized that the algorithms used for the multiply Elastic net and multiply LARS methods, although created in R programming language, have an analogical logical structure. Therefore, they are not included in this paper.

## 3. Results

This chapter presents the results of image reconstruction based on designed numerical models and laboratory measurements. Data analysis is an important part of the diagnosis of the process based on tomography. Knowledge of the process can make the image reconstruction better. Inside the tested object, as its cross-section, a mesh of finite elements is generated. As a result of the calculations it obtains a reconstructed image. The inverse problem was solved using both deterministic and machine learning methods.

**Figure 8:**
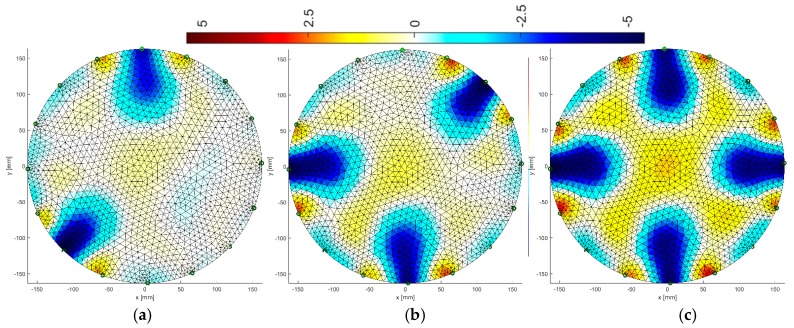
Image reconstruction for 16 measurement electrodes by the Gauss-Newton method with Laplace regularization: (**a**) 2 objects, (**b**) 3 objects, (**c**) 4 objects.

**Figure 9:**
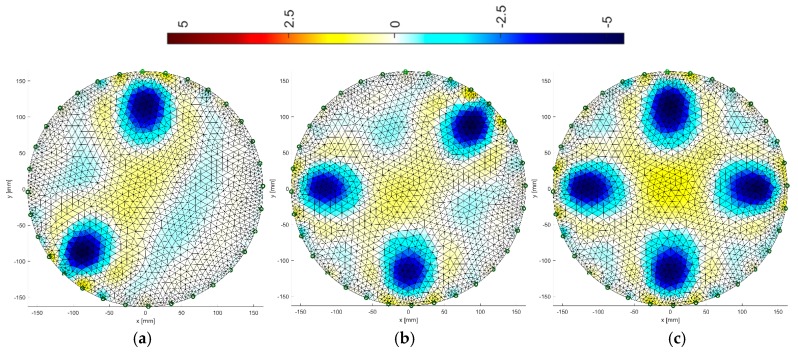
Image reconstruction for 32 measurement electrodes by the Gauss-Newton method with Laplace regularization: (**a**) 2 objects, (**b**) 3 objects, (**c**) 4 objects.

**Figure 10:**
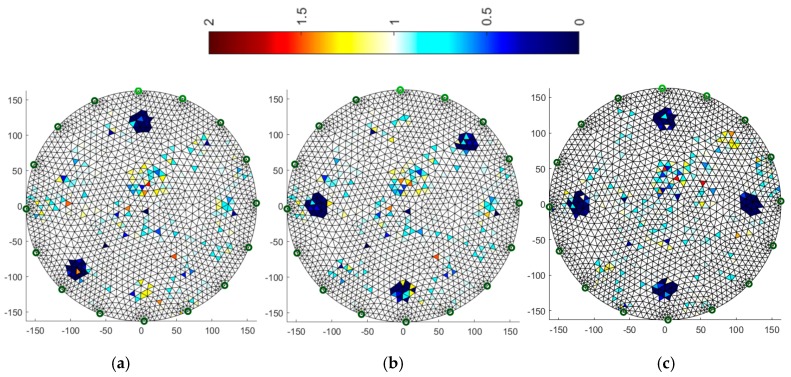
Image reconstruction for 16 measurement electrodes by Multiply Neural Networks: (**a**) 2 objects, (**b**) 3 objects, (**c**) 4 objects.

**Figure 11:**
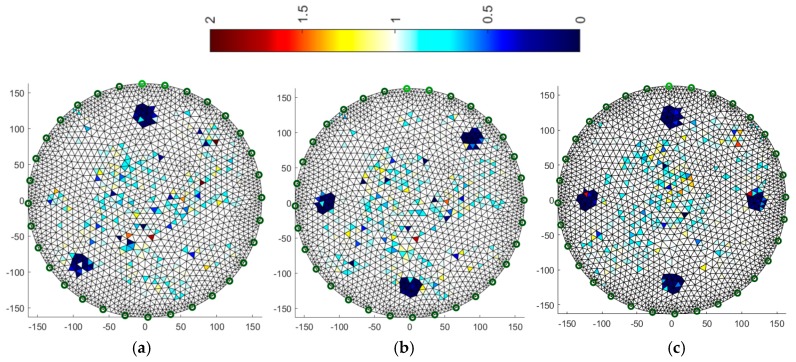
Image reconstruction for 32 measurement electrodes by Multiply Neural Networks: (**a**) 2 objects, (**b**) 3 objects, (**c**) 4 objects.

**Figure 12:**
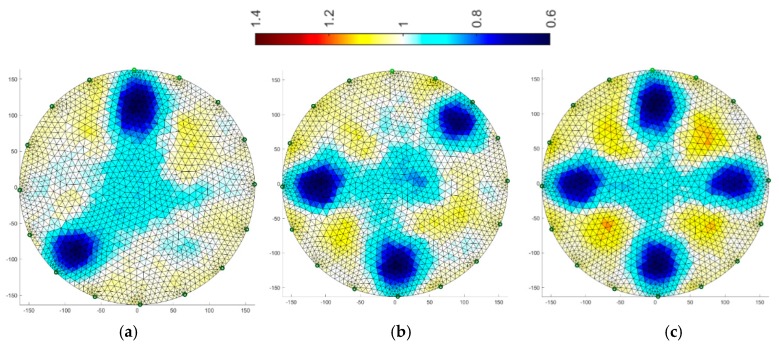
Image reconstruction for 16 measurement electrodes by multiply LARS: (**a**) 2 objects, (**b**) 3 objects, (**c**) 4 objects.

**Figure 13:**
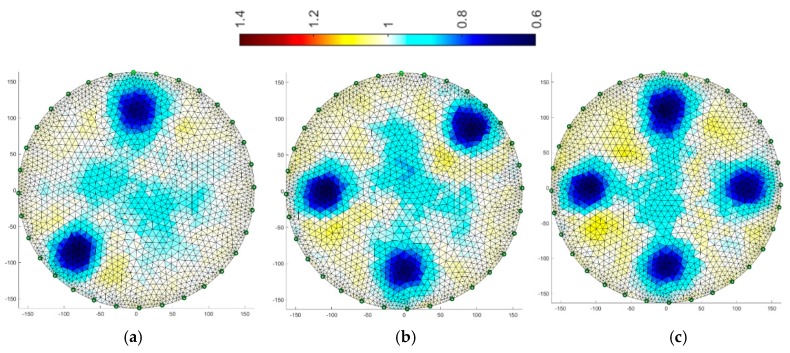
Image reconstruction for 32 measurement electrodes by multiply LARS: (**a**) 2 objects, (**b**) 3 objects, (**c**) 4 objects.

**Figure 14:**
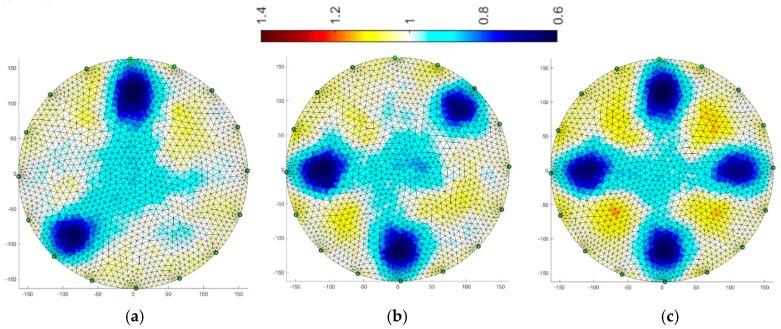
Image reconstruction for 16 measurement electrodes by multiply Elastic net: (**a**) 2 objects, (**b**) 3 objects, (**c**) 4 objects.

**Figure 15:**
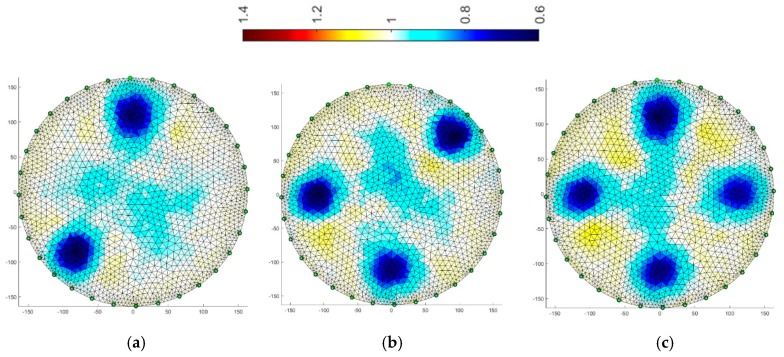
Image reconstruction for 32 measurement electrodes by multiply Elastic net: (**a**) 2 objects, (**b**) 3 objects, (**c**) 4 objects.

Figure 8, Figure 9, Figure 10, Figure 11, Figure 12, Figure 13, Figure 14 and Figure 15 present the results of reconstruction of images based on laboratory measurements of the examined objects. These are not reconstructions based on artificial measurements obtained from a simulation generator. The reconstructions presented below are the result of real measurements generated using a physical model (Figure 3). They contain natural noises and other imperfections, caused by disturbances of the EIT system and the measurement process. As a result, the tomographic images presented below constitute an appropriate comparative basis, enabling objective evaluation of individual reconstruction EIT algorithms.

The systems with 16 and 32 electrodes were used here. Previous research proves that deterministic methods effectively reconstruct the image based on real measurements. The results obtained using multiply neural networks depend primarily on the quality of the training set. In the presented experiments, the data set for ANN was 10 times larger than for LARS or Elastic net and included more cases both in terms of the number of objects (tubes) and their distribution. It is possible that this fact caused the higher quality of the ANN reconstruction. The multiply LARS method is quite sensitive, while multiply Elastic net is quite universal, because by selecting the appropriate regularization parameters you can get enough good reconstructions on the actual data.

All reconstructions presented in this section refer to the three variants of tube arrangements presented in Section 2.2 (Figure 4). Reconstructions were obtained on the basis of test cases generated using the appropriate script. The white color means background. The objects are blue. The colors of the image are correlated with the conductance of the area represented by particular points on the mesh of a given cross-section. All reconstructed images were not improved by data filtering or denoising.

### 3.1. Gauss-Newton Method

The Gauss-Newton with Laplace regularization method was used to reconstruct the image in the electrical tomography for the 16 and 32 electrode systems (Figure 8 and Figure 9). The reconstructions were obtained using the Gauss-Newton method using Laplace regularization. The numerical algorithm operated on a differential basis. So, in this case, we solve the inverse problem after the first iteration. The regularization parameter was 0.08. The reconstructed images illustrate variants with 2, 3 and 4 artifacts.

By comparing the reconstructive images from Figure 8 and Figure 9 to Figure 4 from Section 2.2, it can be seen that the visual mapping of the position of the objects is correct, but their diameters are larger than in the reference images. The background noise is also visible because it should be uniformly white. There are also significant differences in the quality of images obtained from 16 and 32 electrode systems. The use of 32 electrodes gives much better results in this case.

### 3.2. Multiply Neural Networks

Image reconstruction in the case of multiply neural networks depends largely on the training set. An interesting observation is that the use of 32 electrodes (Figure 11) with respect to 16 (Figure 10) does not affect the visual quality of the imaging.

Comparing the reconstructive images of Figure 10 and Figure 11 to Figure 4, it can be seen that the visual representation of the position and also the size of the objects is clearly better than for the Gauss-Newton method. Noise is visible, but it is relatively small and rather point-like. 

### 3.3. Multiply LARS

Another algorithm was based on the multiply LARS method. A training set of 5000 elements was used here. In this case, the obtained results for a system with 16 electrodes (Figure 12) are slightly worse than for a system with 32 electrodes (Figure 13). The key element in this method is the separation of a group of independent measurements. The visual mapping of the position of the objects is correct, but their diameters are larger than in the reference images.

### 3.4. Multiply Elastic Net

The final algorithm is multiply Elastic net. It is more universal due to its character and gives quite precise results. 

The same training set was used as for the previous method. Reconstructions for systems with 16 and 32 electrodes are shown in Figure 14 and Figure 15, respectively. The diameters of inclusions are larger than the reference ones, however, the two-fold increase in the number of electrodes gives significantly better results. The accuracy of the mapping increases and the amount of background noise decreases.

### 3.5. Comparison of Image Reconstructions

Visual comparison of individual methods (Gauss-Newton, multiply Neural Networks, multiply LARS and multiply Elastic net) is not very precise. In order to increase the fairness of the comparison, special indicators calculated using mathematical methods were applied. To make this possible using the simulation method, reference images (vectors) were designed for all 6 cases examined. The dimensions of the physical model presented in Section 2.2 were used for this purpose. It is quite easy because in all tested variants the pixels of the background are white (value 1) and the identified objects (tubes) have a dark blue color (value 0) on the cross-section.

In order to compare the methods, the following evaluation metrics were used: Mean Squared Error (MSE), Relative Image Error (RIE), Image Correlation Coefficient (ICC) and the Expected Time of Image Reconstruction expressed in seconds. MSE is evaluated according to (13)

(13) $$\mathrm{MSE}=\frac{1}{n}\sum_{i=1}^{n}{\left({y^{\prime }}_{i}-y^{*}{}_{i}\right)}^{2}$$

where n is the output image resolution, ${y^{\prime }}_{i}$ is the value of *i* reference pixel, and $y^{*}{}_{i}$ is the value of *i* reconstructed pixel.

RIE is evaluated according to (14).

(14) $$\mathrm{RIE}=\frac{\left\|y^{\prime }-y^{*}\right\|}{\left\|y^{\prime }\right\|}$$

ICC is evaluated according to (15).

(15) $$\mathrm{ICC}=\frac{\sum_{i=1}^{n}\left(y_{i}^{*}-{\bar{y}}^{*}\right)\left({y^{\prime }}_{i}-{\bar{y}}^{\prime }\right)}{\sqrt{\sum_{i=1}^{n}{\left(y_{i}^{*}-{\bar{y}}^{*}\right)}^{2}\sum_{i=1}^{n}{\left(y_{i}^{\prime }-{\bar{y}}^{\prime }\right)}^{2}}}$$

where: ${\bar{y}}^{\prime }$ is the mean value for reference pixels; ${\bar{y}}^{*}$ is the mean value for reconstructed pixels.

The smaller the MSE and RIE, the better the reconstruction quality. ICC is vice versa, the closer to 1, the better the reconstruction.

Table 2 presents the analysis of the quality of image reconstruction for individual methods. The column headers contain information about the number of electrodes in the measurement system (E16 or E32) and the number of hidden objects (O2, O3, O4). For example, E16_O2 means a measuring system with 16 electrodes applied to a case with 2 objects hidden inside the tested tank.

Analyzing the indicators in Table 2, it can be noticed that for all 6 tested variants and 3 indicators the best quality of reconstruction was obtained with the multiply ANN. The rest of the methods differ in relation to both variants and indicators. For example, in the reconstruction of E16_O2, the best MSE was with Elastic net (MSE = 0.0111), the best RIE was for LARS (RIE = 0.1053) and the best ICC was for Gauss-Newton with Laplace regularization (ICC = 0.5290). So, it is impossible to unambiguously determine the best method from the multiply Elastic net, multiply LARS and Gauss-Newton, but indisputably, the best results were obtained using ANN in the tested cases. At the same time, it can be noticed that multiply ANN is the slowest method among all the tested algorithms, while the fastest methods proved to be multiply Elastic net and multiply LARS.

The learning times of tomographic algorithms based on the analyzed methods belonging to the machine learning group depend on a lot of factors. For example, the training time of the multiply ANN for 16 electrodes, by employing one central processing unit (CPU) core took about 27 hours but with 24 cores it was 4.4 hours. The multiply Elastic net and multiply LARS methods are much faster than multiply ANN. With one core it was about 90 seconds and with 24 cores the training time was about 25 seconds. In the case of 32 electrodes, the training times are about 13% longer for ANN and 5 times longer for Elastic net and LARS.

## 4. Conclusions

The monitoring systems are aimed at automation, analysis and optimization of technological processes using industrial tomography, which allows for analysis of processes taking place in a facility without interfering with its interior. Such solutions enable better understanding and monitoring of industrial processes and facilitate process control in real time. The collected information is processed by an algorithm that reconstructs the image. This type of tomography is characterized by a relatively low image resolution. Difficulties in obtaining high resolution result mainly from a limited number of measurements, non-linear current flow through a given medium and too-low sensitivity of measured voltages depending on changes in conductivity in the area. The main challenge in this area is to design precise measuring devices and algorithms for image reconstruction. 

Data analysis is an important part of the diagnosis of the process based on tomography. The inverse problem is the process of optimization, identification or synthesis, in which the parameters describing the field are determined based on the possession of information relevant to this field. Such problems are difficult to analyze. They do not have unambiguous solutions and are misunderstood due to too little or too much information. Knowledge of the process can make image reconstruction more resistant to incomplete information. In the article, the authors used the deterministic method based on the Gauss-Newton method with Laplace regularization as a reference for the selected machine learning methods.

In the process based on electrical tomography, there is no ideal method for reconstructing and analyzing data. Methods and models need to be properly selected depending on the specificity of the problem that needs to be solved. Deterministic methods are usually more awkward with many hidden objects requiring reconstruction. Multiply Neural Networks give better results, but this is mostly dependent on the quantity and quality of the training set. With a large training set, especially for smaller objects, they are really effective. Machine learning methods based on multiply LARS, especially multiply Elastic net, seem to be less accurate, especially for real measurement data, but they are much faster than multiply ANN. The disadvantage of ANN is the long training time and the relatively long reconstruction time. The obtained results were illustrated graphically, which gives the possibility of visual analysis of the processes taking place inside the object, as well as with the use of numerical indicators. The proposed algorithms and gained knowledge should bring benefits to various economic and industrial sectors.

Further works will be focused on improving the methods of image reconstruction using deep learning and the development of measuring devices for both electrical tomography and ultrasound tomography. 

## Figures and Tables

**Table 1:** Comparison of the neural networks with 1 and 10 responses.

Quality Indicators for Testing Set	ANN Type		
	96—10—1	96—10—10	96—20—10
$\bar{MSE}$	0.0069	0.0087	0.0086
$\bar{R}$	0.7548	0.6994	0.6897

**Table 2:** Comparison of image reconstruction indicators.

Methods	Evaluation Metrics	Tested Cases					
		E16_O2	E16_O3	E16_O4	E32_O2	E32_O3	E32_O4
**ANN**	MSE	0.0074	0.0086	0.0076	0.0060	0.0061	0.0058
	RIE	0.0869	0.0936	0.0886	0.0782	0.0785	0.0771
	ICC	0.7356	0.7371	0.8218	0.7484	0.8163	0.7946
	Expected time of image reconstruction [s]	0.1501	0.1578	0.1574	0.2776	0.2785	0.2787
**Elastic net**	MSE	0.0111	0.0148	0.0197	0.0081	0.0131	0.0174
	RIE	0.2466	0.3499	0.3451	0.2120	0.2661	0.3300
	ICC	0.5024	0.4651	0.4535	0.5090	0.4785	0.4702
	Expected time of image reconstruction [s]	0.00062	0.00066	0.00071	0.0013	0.0014	0.0014
**LARS**	MSE	0.0115	0.0153	0.0203	0.0074	0.0121	0.0160
	RIE	0.1053	0.1216	0.1402	0.0871	0.1113	0.1280
	ICC	0.4658	0.4586	0.4438	0.5261	0.5072	0.5082
	Expected time of image reconstruction [s]	0.00041	0.00095	0.00092	0.0019	0.0018	0.0018
**Gauss-Newton with Laplace regulari-zation**	MSE	0.0199	0.0267	0.0351	0.0110	0.0164	0.0225
	RIE	0.1661	0.2524	0.3415	0.1563	0.1755	0.2402
	ICC	0.5290	0.4643	0.4181	0.5853	0.5984	0.5412
	Expected time of image reconstruction [s]	0.01248	0.01010	0.00940	0.01159	0.01229	0.01197
